# Genome-Wide Association Studies of Virulent and Avirulent *Haemophilus parasuis* Serotype 4 Strains

**DOI:** 10.1128/genomeA.00884-14

**Published:** 2014-09-04

**Authors:** Paulraj K. Lawrence, Brittanny L. Wiener, Tammy Kolander-Bremer, Russell F. Bey, Douglas L. Stine, Weerayuth Kittichotirat, Roger E. Bumgarner

**Affiliations:** a Newport Laboratories, Inc., Worthington, Minnesota, USA; b Systems Biology and Bioinformatics Research Group, Pilot Plant, Development and Training Institute, King Mongkut’s University of Technology Thonburi, Bangkhuntien, Bangkok, Thailand; c Department of Microbiology, University of Washington, Seattle, Washington, USA

## Abstract

*Haemophilus parasuis* is a normal commensal of the upper respiratory tract of healthy pigs. However, in conjunction with stress and/or viral infections, or in immunocompromised animals, *H. parasuis* can transform into a pathogen causing Glasser’s disease, which is typically characterized by fibrinous polyserositis, polyarthritis, meningitis, and sometimes acute pneumonia and septicemia. *H. parasuis* serotype 5 is highly virulent and more frequently isolated from respiratory and systemic infection in pigs. Recently Newport Laboratories isolated highly virulent *H. parasuis* serotype 4 strains from the tissues of diseased pigs. This study was undertaken to identify the genes responsible for *H. parasuis* serotype 4 virulence. To achieve this objective we performed genome-wide association studies (GWAS) across two virulent and three avirulent *H. parasuis* serotype 4 strains.

## GENOME ANNOUNCEMENT

*Haemophilus parasuis* is Gram-negative, nonmotile, nonhemolytic, pleomorphic and nicotinamide adenine dinucleotide (NAD)-dependent bacterium (1, 2). It is a normal commensal of the upper respiratory tract of healthy pigs (3, 4). However, during stress in immunocompromised animals, *H. parasuis* transforms into a pathogen and causes Glasser’s disease, which is typically characterized by fibrinous polyserositis, polyarthritis, meningitis, and sometimes acute pneumonia and septicemia (3–5). This disease is one of the primary causes of morbidity and mortality in the U.S. and swine industry worldwide, resulting in substantial economic losses.

To date, 15 different serotypes of *H. parasuis* have been identified based on the presence of heat stable antigens and gel diffusion tests. However, a high percentage of the field isolates are nontypable (6). Among the 15 serotypes, *H. parasuis* serotype 5 is more frequently isolated from respiratory and systemic infections in pigs thus, appears to be highly virulent (7, 8). During the past few years, Newport Laboratories has observed an increase in the number of cases of *H. parasuis* serotype 4 infections (ST4) in the U.S. Surveys in the past have indicated that *H. parasuis* serotype 4 is an emerging pathogen in the U.S. swine population with a potential to cause serious outbreaks (9). In an attempt to understand the differences between virulent and avirulent *H. parasuis* ST4, we performed a genome-wide association studies (GWAS) across two virulent and three avirulent strains.

*H. parasuis* ST4 virulent strains (ST4-1 and ST4-2) and the avirulent strains (HPS9, HPS10, and HPS11) were cultured on Trypticase Soy Agar (TSA) plates supplemented with 5% bovine serum and 10 mg/ml nicotinamide adenine dinucleotide (NAD). Bacterial cells were scrapped from the plates, pelleted, and the genomic DNA was extracted using a bacterial genomic DNA isolation kit (Edge Biosystems). The genomic DNA library was generated by a TruSeq Library preparation kit (Illumina, Inc.) following the manufacturer’s recommendation and sequenced on an Illumina MiSeq to obtain 150 bp paired-end (PE) reads. Whole-genome sequencing was performed at the University of Minnesota Genomics Center and University of Washington. Serotype identity of all the *H. parasuis* strains isolated in this study was confirmed by the Department of Veterinary Medicine, University of Montréal.

GWAS identified phage and hypothetical proteins unique to each isolate and each phenotypic group. The virulent strains encode exclusive proteins such as maltose acetyltransferase (HPS41_00460, HPS42_04310), peptidoglycan-binding protein LysM (HPS41_01895, HPS42_11245), peptidase S14 (HPS41_08645, HPS42_07140), guanine-1-methyltransferase (HPS41_09075, HPS42_02465), S/T phosphatase (HPS41_00405, HPS42_10265), and a tRNA-Arg (HPS41_00420, HPS42_10250). It is striking to note that ST4-1 and ST4-2 lack silalytransferase (CMP-N-acetylneuraminate-beta-galactosamide- alpha-2, 3-sialyltransferase), an enzyme critical for sialylation of terminal N-acetyllactosamine trisaccharide during lipo-oligosaccharide (LOS) synthesis. Lacking sialyltransferase could reduce the amount of sialic acid on the cell surface and can cause hypersensitive reactions in the host. In contrast, all avirulent strains encode sialyltransferase (HPS9_01535, HPSM10_00215, HPSM11_05065). Few of the functional proteins exclusive to avirulent strains include; preprotein translocase subunit SecG (HPS9_01510, HPSM10_08005, HPSM11_00325), metal-dependent hydrolase (HPS9_02995, HPS10_04690, HPS11_08015), cell filamentation protein Fic (HPS9_03915, HPSM10_10285, HPSM11_09710), and VbhA antitoxin and related proteins (HPS9_03920, HPS10_10280, HPS11_09715).

### Nucleotide sequence accession numbers.

This whole-genome shotgun project has been deposited at DDBJ/EMBL/GenBank under the accession numbers JJNQ00000000 (ST4-1), JJNR00000000 (ST4-2), JDSN00000000 (HPS9), JDSO00000000 (HPS10), and JDSP00000000 (HPS11). The versions described in this paper are JJNQ00000000.1, JJNR00000000.1, JDSN00000000.1, JDSO00000000.1, and JDSP00000000.1, respectively.
